# 非小细胞肺癌切除组织中常用免疫组织化学指标尚不能预测远期生存

**DOI:** 10.3779/j.issn.1009-3419.2016.03.05

**Published:** 2016-03-20

**Authors:** 潞艳 申, 晓征 康, 宇 孙, 浩 付, 亮 戴, 万璞 闫, 克能 陈

**Affiliations:** 1 100142 北京，北京大学肿瘤医院暨恶性肿瘤发病机制及转化研究教育部重点实验室，胸外一科 Key Laboratory of Carcinogenesis and Translational Research (Ministry of Education), Department of Thoracic Surgery Ⅰ, Beijing 100142, China; 2 100142 北京，北京大学肿瘤医院暨恶性肿瘤发病机制及转化研究教育部重点实验室，病理科 Department of Pathology, Peking University Cancer Hospital & Institute, Beijing 100142, China

**Keywords:** 肺肿瘤, 预后, 免疫组织化学, Lung neoplasms, Prognosis, Immunohistochemistry

## Abstract

**背景与目的:**

在非小细胞肺癌（non-small cell lung cancer, NSCLC）切除组织中寻找可预测远期生存的免疫组化指标一直备受关注。本研究旨在回顾评价单中心历史上曾选用的免疫组化指标与NSCLC预后之间的关系。

**方法:**

2008年-2013年我院单一手术组切除NSCLC 722例，选用的免疫组化指标共12个，在随访良好的前瞻性数据基础上，行单因素生存分析及多因素风险回归模型评价这些指标的表达在NSCLC切除生存中的意义。

**结果:**

曾选用的12个免疫组化分子分别为：血小板衍生生长因子受体（platelet-derived growth factor receptor, PDGFR）（*n*=460）、切除修复交叉互补1（excision repair cross complementing 1, ERCC1）（*n*=461）、表皮生长因子受体（epithelial growth factor receptor, EGFR）（*n*=460）、人血管内皮生长因子受体3（vascular endothelial growth factor receptor 3, VEGFR3）（*n*=451）、NM23（*n*=359）、MRP（*n*=351）、P170（*n*=353）、TS（*n*=431）、Tubulin（*n*=307）、核糖核苷酸还原酶M1（ribonucleotide reductase M1, RRM1）（*n*=381）、环氧酶2（cyclooxygenase 2, COX2）（*n*=364）和TOPII（*n*=235）。单因素分析显示仅有VEGFR3的表达与生存有关，阳性表达者与阴性表达者的5年生存率分别为77.6%与65.0%（*P*=0.042）。但多因素分析表明VEGFR3不是NSCLC独立的预后因素。

**结论:**

本组所选用的免疫组织化学指标不能预测切除后的NSCLC患者的生存。

## Abstract (en)

**Background and objective:**

It has been drawn much attention to identify the molecular markers by immunohistochemistry (IHC) for evaluating the prognosis of non-small cell lung cancer (NSCLC) following resection. The aim of this study is to retrospectively associate ever tested IHC markers and prognosis of NSCLC after resection.

**Methods:**

A total of 722 NSCLC patients underwent surgery by single surgeon team from 2008 to 2013. Twelve molecular markers had been examined by IHC and the staining signals was re-scored with unified standard. Survival analysis by univariate and multivariate was carried out to assess the significance of these markers in prognosis of NSCLC in our prospective database with strict follow-up.

**Results:**

The following twelve IHC markers had been tested between 2008 and 2013, including platelet-derived growth factor receptor (PDGFR)(*n*=124), excision repair cross complementing 1 (ERCC1)(*n*=124), epithelial growth factor receptor (EGFR)(*n*=131), vascular endothelial growth factor receptor 3 (VEGFR3)(*n*=142), NM23 (*n*=129), MRP (*n*=109), P170 (*n*=104), TS (*n*=143), Tubulin (*n*=133), ribonucleotide reductase M1 (RRM1)(*n*=131), ribonucleotide reductase M1 (COX2)(*n*=138), and TOPII (*n*=127). Only VEGFR3 expression was correlated with prognosis of the patients by univariate analysis, with 5-yrs survival rate being 77.6% and 65.0% (positive vs. negative) respectively (*P*=0.042). However, VEGFR3 was not an independent prognostic factor for this series of NSCLC patients in multivariate analysis.

**Conclusion:**

These twelve IHC markers could not predict prognosis of NSCLC patients after surgery in our series.

以免疫组织化学手段检测非小细胞肺癌（non-small cell lung cancer, NSCLC）切除标本中的相关蛋白用于组织分类、治疗选择及预后判断，似乎已广泛见诸于NSCLC的临床实践。然而，回顾各主要临床指南及主要文献却发现在诊断方面较为成熟的指标仅有鉴别鳞癌（P63、P40、CK5/6）与腺癌（TTF-1、Napsin A、CK7）的少数指标^[1]^；用于治疗选择的也仅包括表皮生长因子受体（epithelial growth factor receptor, EGFR）、间变性淋巴瘤激酶（anaplastic lymphoma kinase, ALK）、MET、ROS-1及KRAS等几个为数不多的指标^[2]^；更多的免疫组化分子则是临床尝试用于判断NSCLC预后的研究。相信国内各家医院随着这一领域的研究进展与变迁，也曾选用过许多不同的免疫组化分子标记物，这些分子种类繁多，对预后影响的结果众说纷纭，使临床应用产生过一些混乱。本研究就我院2008年-2013年在NSCLC中曾选用的免疫组化分子作一总结，尤其是这些分子与预后的关系在我们单医生组前瞻性数据库中作了验证，供大家参考。

## 1 资料与方法

### 1.1 患者来源

北京大学肿瘤医院胸外科单一手术组（陈克能）2008年1月-2013年9月行根治性手术治疗NSCLC患者共计722例。病理分期采用国际抗癌联盟（Union for International Cancer Control, UICC）/美国癌症联合会（American Joint Committee on Cancer, AJCC）第七版肿瘤-淋巴结-转移（tumor-node-metastasis, TNM）分期系统^[3]^；病理分型采用2004年世界卫生组织（World Health Organization, WHO）分型^[4]^；手术方式包括：肺叶切除术（*n*=598）、双肺叶切除术（*n*=39）、全肺切除术（*n*=31）、袖式成型术（*n*=10）及其他（*n*=44）。

### 1.2 免疫组化指标

共有12个指标在不同时期被用于检测，分别是血小板衍生生长因子受体（platelet-derived growth factor receptor, PDGFR）、切除修复交叉互补1（excision repair cross complementing 1, ERCC1）、表皮生长因子受体（epithelial growth factor receptor, EGFR）、人血管内皮生长因子受体3（vascular endothelial growth factor receptor 3, VEGFR3）、NM23、MRP、P170、TS、Tubulin、核糖核苷酸还原酶M1（ribonucleotide reductase M1, RRM1）、环氧酶2（cyclooxygenase 2, COX2）和TOPII。

### 1.3 免疫组化方法及判读标准

由于该研究为回顾性研究，历经各种免疫组化方法，因此各指标的具体染色方法及抗体来源等均予以忽略。但对再次判读的方法做了规定：由两位不了解临床病理资料及研究内容的病理科医生采用盲法判读。以阳性对照及阴性对照控制反应体系质量。分别从细胞膜、细胞质、细胞核3方面判断目标蛋白在细胞内的分布特征。每张切片随机选择有代表性的10个高倍视野（×400），连续计数视野下染色阳性的肿瘤细胞。按照染色强度和染色面积（阳性细胞所占构成比）进行评分，最后将两者相乘。无着色为0级计0分，浅黄色为1级计1分，棕黄色为2级计2分，棕褐色为3级计3分；染色面积评分如下：无着色为0分，≤30%计1分，30%-60%计2分， > 60%计3分，两者相乘最终将得到0分、1分、2分、3分、4分、6分、9分七组数据。

### 1.4 生存随访信息

本研究组自2000年始建立了单一手术组的NSCLC前瞻性数据库，其中随访原则为：术后2年内每3个月随访检查1次，2年-5年内每6个月随访检查1次，5年后每年随访检查1次。全组生存计算为总生存随访，全病因死亡。随访起点为手术日，终点为死亡日期，末次随访时间为2015年8月。研究队列随访主要为门诊随访，达到80.0%，电话或书信随访仅占10.6%。门诊随访内容：记录症状学、检查包括血液学检查、肿瘤标记物、胸部增强计算机断层扫描（computed tomography, CT）、头颅增强磁共振成像（magnetic resonance imaging, MRI）、颈、腹部超声、全身骨扫描检查，2010年后部分患者有正电子发射型计算机断层显像（positron emission computed tomography, PET-CT）检查。全组中位随访时间为36.1个月，存活患者中位随访时间为39.6个月。全组死亡患者117例，存活患者537例，失访患者68例，全组随访率达90.6%。

### 1.5 统计学方法

采用SPSS 18.0软件进行统计学分析。采取卡方检验比较NSCLC各指标表达与各临床病理因素之间的关系。采取*Kaplan*-*Meier*生存曲线及*Log*-*rank*检验分析判断各指标表达在NSCLC患者生存间的关系。采取*Cox*风险比例模型分析NSCLC患者的独立预后因素，*P*＜0.05为差异有统计学意义。

## 2 结果

### 2.1 12种免疫组化指标的判读结果

722例NSCLC患者中，12种免疫组化指标染色的病例数分别为PDGFR（*n*=460）、ERCC1（*n*=461）、EGFR（*n*=460）、VEGFR3（*n*=451）、NM23（*n*=359）、MRP（*n*=351）、P170（*n*=353）、TS（*n*=431）、Tubulin（*n*=307）、RRM1（*n*=381）、COX2（*n*=364）、TOPII（*n*=235）（表 1）。12种指标的染色结果按照染色面积及染色强度的乘积计算得分，分为0分、1分、2分、3分、4分、6分、9分，不同分值的分布情况如图 1所示。

**1:** 免疫组化指标染色情况与患者一般临床病理资料之间的关系 Association between biomarkers staining and tumor characteristics of patients with NSCLC

Item (*n*=722)	IHC status	*n*(%)	Neoadj [*n*(%)]		*P*	Histology [*n*(%)]			*P*	Stage [*n*(%)]			*P*
			0	1		ADC	SCC	Other		Ⅰ	Ⅱ	Ⅲ	
PDGFR (*n*=460)	Positive	363 (79)	300 (78)	63 (82)	0.579	269 (82)	82 (73)	12 (67)	0.065	228 (78)	73 (82)	59 (80)	0.684
	Negative	97 (21)	83 (22)	14 (18)		61 (18)	30 (27)	6 (33)		65 (22)	16 (18)	15 (20)	
ERCC1 (*n*=461)	Positive	304 (66)	250 (65)	54 (70)	0.294	149 (45)	56 (50)	5 (28)	0.22	143 (49)	38 (42)	27 (36)	0.128
	Negative	156 (34)	133 (35)	23 (30)		182 (55)	56 (50)	13 (72)		150 (51)	52 (58)	47 (64)	
EGFR (*n*=460)	Positive	304 (66)	250 (65)	54 (70)	0.479	234 (71)	63 (56)	7 (39)	0.001	202 (69)	56 (63)	42 (58)	0.161
	Negative	156 (34)	133 (35)	23 (30)		96 (29)	49 (44)	11 (61)		92 (31)	33 (37)	31 (42)	
VEGFR3 (*n*=451)	Positive	368 (82)	313 (83)	55 (72)	0.018	279 (86)	81 (74)	8 (53)	0.001	242 (84)	69 (79)	53 (73)	0.061
	Negative	83 (18)	62 (17)	21 (28)		47 (14)	29 (26)	7 (47)		45 (16)	18 (21)	20 (27)	
NM23 (*n*=359)	Positive	323 (90)	274 (90)	49 (91)	0.898	245 (92)	70 (84)	8 (73)	0.015	221 (91)	57 (92)	42 (82)	0.152
	Negative	36 (10)	31 (10)	5 (9)		20 (8)	13 (16)	3 (27)		22 (9)	5 (8)	9 (18)	
MRP (*n*=351)	Positive	323 (92)	275 (92)	48 (94)	0.593	240 (92)	75 (94)	8 (80)	0.325	222 (92)	56 (93)	42 (89)	0.745
	Negative	28 (8)	25 (8)	3 (6)		21 (8)	5 (6)	2 (20)		19 (8)	4 (7)	5 (11)	
P170 (*n*=353)	Positive	212 (60)	186 (62)	26 (49)	0.083	170 (65)	37 (45)	5 (50)	0.003	147 (62)	36 (58)	27 (55)	0.666
	Negative	141 (40)	114 (38)	27 (51)		91 (35)	45 (55)	5 (50)		92 (38)	26 (42)	22 (45)	
TS (*n*=431)	Positive	91 (21)	75 (21)	16 (22)	0.678	61 (19)	22 (21)	8 (57)	0.004	55 (20)	23 (28)	13 (19)	0.283
	Negative	340 (79)	285 (79)	55 (78)		253 (81)	81 (79)	6 (43)		222 (80)	60 (72)	54 (81)	
Tubulin (*n*=307)	Positive	251 (82)	209 (82)	42 (82)	0.982	187 (84)	56 (76)	8 (73)	0.19	163 (86)	49 (75)	35 (73)	0.043
	Negative	56 (18)	47 (18)	9 (18)		35 (16)	18 (24)	3 (27)		27 (14)	16 (25)	13 (27)	
RRM1 (*n*=381)	Positive	205 (54)	171 (53)	34 (58)	0.643	160 (56)	39 (45)	6 (50)	0.179	144 (56)	32 (48)	25 (45)	0.238
	Negative	176 (46)	151 (47)	25 (42)		123 (44)	47 (55)	6 (50)		112 (44)	34 (52)	30 (55)	
COX2 (*n*=364)	Positive	262 (72)	231 (75)	31 (55)	0.003	201 (74)	54 (66)	7 (58)	0.204	169 (69)	54 (86)	38 (73)	0.026
	Negative	102 (28)	77 (25)	25 (45)		69 (26)	28 (34)	5 (42)		77 (31)	9 (14)	14 (27)	
TOPⅡ (*n*=235)	Positive	208 (89)	175 (89)	33 (87)	0.579	164 (88)	41 (91)	3 (75)	0.423	144 (88)	32 (89)	30 (91)	0.913
	Negative	27 (11)	22 (11)	5 (13)		22 (12)	4 (9)	1 (25)		19 (12)	4 (11)	3 (9)	
NSCLC: non-small cell lung cancer; PDGFR: platelet-derived growth factor receptor; ERCC1: excision repair cross complementing 1; VEGFR3: vascular endothelial growth factor receptor 3; RRM1: ribonucleotide reductase M1; COX2: cyclooxygenase 2; IHC: immunohistochemistry.													

**1:**
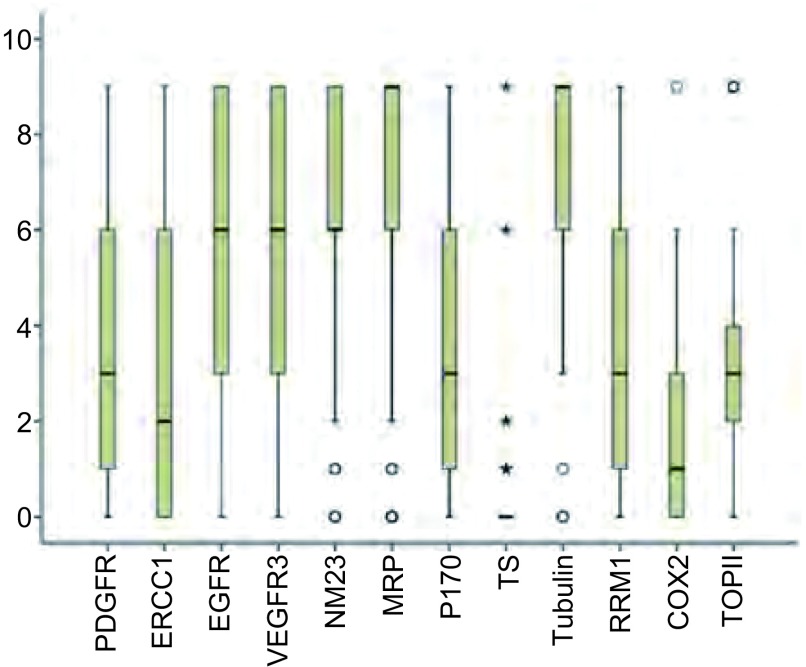
免疫组化指标染色评分的分布情况。o、★代表离散值。 Distribution of immunohistochemistry scores. o, ★ represent discrete values.

### 2.2 12种免疫组化指标在NSCLC组织中的表达与病理特征之间的关系

12种蛋白在NSCLC组织中均有不同程度的表达，表达阳性率分别为：PDGFR为78.9%（363/460）、ERCC1为45.6%（304/461）、EGFR为66.1%（304/460）、VEGFR3为81.6%（368/451）、NM23为90.0%（323/359）、MRP为92.0%（323/351）、P170为60.1%（212/353）、TS为21.1%（91/431）、Tubulin为81.8%（251/307）、RRM1为53.8%（205/381）、COX2为72.0%（262/364）、TOPII为88.5%（208/235）（表 1）。卡方检验分析12个指标的表达与病理分期，组织类型之间的关系，结果发现EGFR、VEGFR3、NM23、P170在腺癌中的阳性表达率明显高于非腺癌，Tubulin在Ⅰ期NSCLC中的阳性表达率明显高于Ⅱ期和Ⅲ期NSCLC（表 1）。

### 2.3 12种免疫组化指标在NSCLC组织中的表达与患者预后之间的关系

单因素生存分析结果显示，在12个指标中仅有VEGFR3的表达与本组患者的总生存明显相关，其中VEGFR3阴性表达者的5年生存率明显低于阳性表达者（65% *vs* 77.6%）（图 2）；其他11种指标的表达与患者的总生存间均无明显相关性（表 2）。但在多因素分析结果显示，VEGFR3不是NSCLC的独立预后因素（表 3）。

**2:**
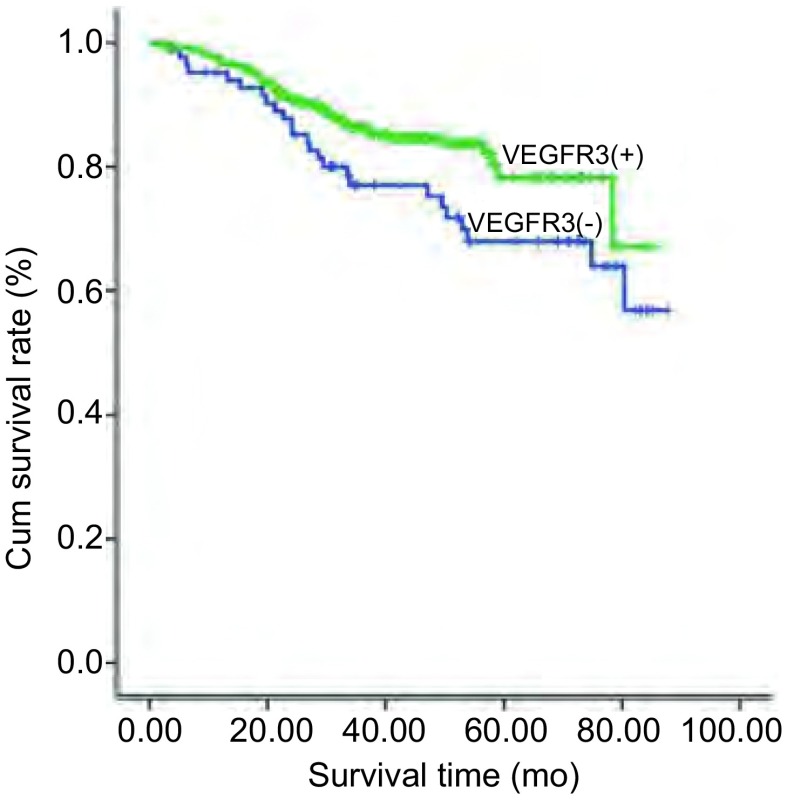
VEGFR3的表达与NSCLC患者预后之间的关系（*n*=451） Association between VEGFR3 expression and prognosis of patients with NSCLC (*n*=451)

**2:** 免疫组化指标的表达水平不同对总生存的影响 Overall survival according to biomarkers expression level

Item	5-yrs survival rate	*P*
VEGFR3-positive	77.6%	0.042
VEGFR3-negative	65.0%	
PDGFR-postitive	74.9%	0.180
PDGFR-negative	64.2%	
ERCC1-postitive	81.0%	0.271
ERCC1-negative	73.0%	
EGFR-postitive	72.0%	0.492
EGFR-negative	78.7%	
NM23-postitive	76.4%	0.505
NM23-negative	85.0%	
MRP-postitive	80.0%	0.718
MRP-negative	90.0%	
P170-postitive	83.0%	0.739
P170-negative	75.8%	
TS-postitive	71.9%	0.058
TS-negative	78.9%	
Tubullin-postitive	80.0%	0.186
Tubullin-negative	66.7%	
RRM1-postitive	85.0%	0.604
RRM1-negative	78.9%	
COX2-postitive	76.0%	0.908
COX2-negative	85.9%	
TOPⅡ-postitive	89.0%	0.412
TOPⅡ-negative	---	

**3:** NSCLC患者*Cox*风险比例模型分析 Independent predictors of disease-free survival time in multivariate analysis

Item	HR	95%CI	*P*
Age (< 61 yr *vs* > 61 yr)	2.244	1.381-3.647	0.001
Gender (male *vs* famale)	0.860	0.500-1.480	0.586
TNM stage			< 0.001
Ⅰ *vs* Ⅲ	0.244	0.141-0.424	< 0.001
Ⅱ *vs* Ⅲ	0.493	0.274-0.888	0.019
Histology			0.470
SCC *vs* ADC	0.864	0.487-1.530	0.615
Other *vs* ADC	1.586	0.624-4.031	0.332
Neoajuvant chemotherapy (no *vs* yes)	1.843	1.071-3.173	0.027
VEGFR3 (positive *vs* positive)	0.750	0.451-1.246	0.267
TNM: tumor-node-metastasis; SCC: squamous cell carcinoma; ADC: adenocarcinoma.			

## 3 讨论

肺癌成功的治疗始于1933年外科切除^[5]^，但直到1959年-1962年才从病理上区别了NSCLC与SCLC，随后确立了SCLC不宜手术的概念^[6]^。至1967年才有了肺鳞癌、肺腺癌的分类^[7]^。但长期以来仍有相当多的NSCLC从HE染色上不能区分鳞癌和腺癌，尤其是在活检标本中不能区分者高达30%-50%，然而随着治疗方法的改进与更新要求我们对NSCLC应该更准确地进行组织分类，尤其是肺腺癌的确认直接关乎治疗手段的选择（化疗、靶向治疗）。因此，2011年确立了P63、P40、TTF-1、Napsin A作为区分NSCLC的除HE染色后又一金标准，尤其是对HE不能分类者是必查项目^[8]^。同时，Ventana法检测ALK在判断是否应用克唑替尼方面起了决定作用^[9]^，应该说免疫组化分子检查已成为当今NSCLC临床不可或缺的检测手段。

回顾各国制定的NSCLC的各种指南，并没有将任何免疫组化分子引入NSCLC的预后判断系统，即便有学者将ERCC1用于判断化疗疗效的指标^[10]^，由于缺乏持续的临床证据，也在随后的实践中停止应用^[11]^。然而，探讨免疫组化分子对NSCLC预后的影响的研究从未停止过，大规模研究可以追溯到2008年，迄今没有得出较统一的认识，究其原因如下：①抗体的来源和质量不一；②染色流程不一；③评分体系不一；④统计方法不一；⑤小样本的研究降低统计效能。正如近期Lindskog等^[12]^在综述中指出的一样，目前关于预测NSCLC预后的免疫组化标记物的研究层出不穷，所涉及的分子繁多，但大量研究样本量较小导致所得到的结论说服力不足；同时缺乏验证数据；即使有少数的研究做了外部验证，结果往往不一致。作者共纳入2008年1月1日-2013年6月30日期间的347篇文献，涉及342种蛋白分子；样本量＜100的研究占三分之一以上，这些研究大多数为单个队列的分析，仅有10项研究进行了外部验证。所涉及的分子中，有168个（49%）在两项以上的研究中被报道，其中至少在两篇报道中结果一致的分子仅有67个。这些分子目前没有一个可用于临床常规工作中。Zhu等^[13]^总结了1987年-2005年关于CCND1在NSCLC患者中的预后意义的15项研究，其中5项研究表明CCND1高表达，预后差；3项研究表明CCND1高表达预后好；其余7项研究都未发现CCD1表达与预后之间的相关性。因此，可以看出免疫组化分子用于判断预后遇到极大的挑战，染色流程的标准化规范，更为严谨的研究设计以及解读方法等都是亟需解决的问题。

除了复习文献外，我们回顾了我院2008年-2013年间NSCLC曾选用的免疫组化分子PDGFR、ERCC1、EGFR、VEGFR3、NM23、MRP、P170、TS、Tubulin、RRM1、COX2、TOPII的染色情况，分析这些指标与NSCLC患者预后之间的关系，发现只有VEGFR3的表达与患者生存相关，VEGFR3阳性表达者的5年生存率高于阴性表达者。关于这一指标已有相关研究报道，Donnem等^[14]^在335例可切除的Ⅰ期-Ⅲa期NSCLC患者的组织芯片中检测了PDGFR-α、PDGFR-β、以及VEGFR-3蛋白水平的表达，进而分析发现肿瘤细胞共表达VEGFR-3和PDGF-B与淋巴结转移呈正相关，且是独立的预后不良因素（HR=4.8, 95%CI: 2.80-8.31, *P*＜0.001）。Andersen等^[15]^在55例可切除的Ⅰ期-Ⅲa期行术后辅助化放疗的患者组织中检测了VEGFR-1、VEGFR-2、VEGFR-3的表达，并分析了他们的表达与患者预后关系，发现仅单因素分析结果显示VEGFR-1、VEGFR-2、VEGFR-3的高表达与患者预后较差相关。我们与以往研究的结果相反，同时我们的结果在多因素分析中也未能证实，这也反映了免疫组化检测预后分子标记物的现状与困境，由于免疫组化固有的缺陷导致目前仍然难以形成规范用于临床。综上所述，无论是以往研究的文献检索还是我们单中心的验证数据均说明目前所采用的免疫组化指标不能预测NSCLC切除后的预后。

本研究存在如下缺陷：①回顾性研究；②时间跨度大，免疫组化指标染色非同一时期完成，试剂不统一，各指标行免疫组化染色的病例数不等，以上缺陷均会影响结果的判读。因此，我们还需慎重解读，且需进一步积累数据。对NSCLC生物学本质的深入理解和寻找新的分子标记物将是未来研究的方向。
